# The Registration of Nurses

**Published:** 1902-06

**Authors:** 


### The Registration of Nurses.

THE New York State Nurses' Association completed its organisation at the annual meeting held in Albany on April 15, 1902. The officers elected are: President, Miss Isabel Merritt, Cherry Valley; 1st vice-president, Miss Julia Bailey, Rochester; 2d vice-president, Miss E. J. Keating, Buffalo; secretary, Miss E. C. Sanford, Rochester; treasurer, Miss Mary Brooks, Saratoga. Trustees: one year, Miss A. C. Maxwell, New York; two years, Miss S. F. Palmer, Rochester; three years, Miss L. L. Dock, New York. Chairman of committees: by-laws, Miss Ida R. Palmer, Albany; legislation, Miss Eva Allerton, Rochester; publication, Miss S. F. Palmer, Rochester; credentials, Miss A. C. Maxwell, New York; finances, Mr. L. B. Sanford, New York.

The society announces that it is ready to consider seriously the question of legislation for registration, which will ultimately place training schools for nurses under the supervision of the regents of the University of the State of New York, establishing thereby a more uniform basis of nursing education in the state, and eventually making trained nursing a recognised profession. This means a great reform, to accomplish which the nurses look to the coöperation of medical journals and medical societies, as well as the support of every intelligent citizen. The practical result of registration will be the protection of the public against imposters, of whom there are scores in every large nursing center, and a gradual raising of the standard of admission to training schools, with a more carefully prepared curriculum of both theoretical and practical instruction; in other words, better women, better nurses, fewer imposters, and a recognised profession for a great army of workers who now have no legal status.

The nurses of Illinois and New Jersey are already organised for this purpose, with Colorado, North Carolina, Pennsylvania and Massachusetts moving forward on similar lines. The movement is also strong in England.

The JOURNAL in its issue for April, 1902, dealt with this subject in its editorial columns, and is pleased to open them again in furtherance of the objects indicated by the organised body of nurses in the State association.

---

THE prevailing high price of beef caused, it is believed, by the action of the socalled beef trust, has led to a discussion relating to the use of other articles of food in substitution for meat. Professor H. W. Wiley, chief chemist of the department of agriculture, has been quoted on this subject as follows:

Without going into the question of price, Indian corn, wheat, rye and rice contain, as far as actual nourishment is concerned, everything essential to supply the waste of the body and give the necessary health and energy to the system, and it is well known that men nourished extensively on cereals are capable of the hardest and most enduring manual labor. Meats are quickly digested, and furnish an abundance of energy soon after consumption, but are not retained in the digestive organism long enough to sustain permanent muscular exertion. On the other hand, cereal foods are more slowly digested, furnish the energy necessary to digestion and the vital functions in a more uniform manner, and are thus better suited to sustain hard manual labor for a long period of time.

The workingmen of the country, Professor Wiley said, should consider this point, and accustom themselves more to the use of cereals in their foods. When properly prepared and served, they are palatable as well as nutritious, and their judicious use will tend to diminish the craving for meat, which, however, it is not advisable to exclude entirely from the diet.

Professor Wiley further said that it seemed probable that the price of meat, now abnormally high, will never again descend to as low a point as that reached a few years ago, and that the condition which now confronts the American people is, therefore, one of considerable permanency, and should be met and considered as such.

---

THE volcanic eruptions in the Windward Islands that occurred May 8, 1902, and during subsequent days, caused the most horrible destruction of human life in recent years. The city of St. Pierre with its 30,000 inhabitants has disappeared from the face of the earth and all of the population in the northern part of St. Vincent, about 1,700 in number, has been killed. Such a marvelous destruction of life and property is difficult to comprehend and pitiable to contemplate. It is to the credit of the American people that contributions of stores, provisions, medicines and money were promptly made in more than adequate quantities to meet the emergency, and that medical officers were sent forward to minister to the suffering survivors.

---

BRIGADIER-GENERAL GEORGE M. STERNBERG, Surgeon-general U. S. Army, will retire from active service June 8, 1902, by reason of age limit. The conspicuous services rendered by General Sternberg receive the commendation of medical men throughout the world. It is announced that congress has taken action looking toward Dr. Sternberg's promotion to the rank of Major General on the date of his retirement. This would be a gratifying recognition of faithful and efficient service, and we earnestly hope that it may be done. As a matter of fact the surgeon-general of the army should rank as a major-general as soon as he takes office, rather than when he leaves it; but even the latter promotion is better than none at all.

---

In another column we publish a review of two volumes of Nothnagel's System of Practical Medicine together with a portrait of Professor Nothnagel. The English translation of this great work, which is publishing by W. B. Saunders & Company, of Philadelphia, will furnish a supply of the best recent literature on medicine.

The publishers have furnished us with a biographical sketch of the distinguished editor, author and teacher, from which we extract the following:

Nothnagel today is at the height of his scientific activity, which dates from the year 1865, when he was appointed Leyden's assistant in Königsberg. In 1867 he was Docent at Breslau; in the years 1872 and 1873 he taught in the capacity of professor at the medical policlinic in Freiburg in Baden; later he was Ordinarius in Jena from 1874 to 1882, when he was called to Vienna. His first celebrated work, Handbuch der Arzneimittellehre, appeared in 1870. This was followed by two essays replete with original observations: Topische Diagnostik der Gehirnkrankheiten, in 1879, and Beiträge zur Physiologie und Pathalogie des Darmes, in 1884. Experimental investigations of the highest importance are contained in these publications, as well as in shorter compositions on the brain, the effect of lightning, temperature-sense, vasomotor neuroses, convulsions, Addison's disease, and the cardiac regulating mechanism. More recently, Nothnagel has written on two modern subjects, Compensatory Processes in Pathologic Conditions and Vascular Pain. Since the year 1894 he has been engaged in editing the great Specielle Pathologie und Therapie, for which he himself wrote the volume on the Diseases of the Intestine and Peritoneum. This magnificent work is now being translated into English and will be published by W. B. Saunders & Company, of Philadelphia.

---

Governor-General Leonard Wood on May 20, 1902, relinquished the control of the United States over the island of Cuba to President T. Estrada Palma, lowering with his own hand the United States flag that has for more than three years floated over the government palace at Havana, and raising in its place the colors of "Cuba Libre."

Marvelous changes have been wrought in the island during Governor Wood's administration. Schools have been established, courts of justice reformed, sewers built, streets paved, and police administration put upon the highest plane of efficiency and economy. But best of all, the sanitary condition of the entire island and especially of the two chief cities has been brought up to a standard that compares favorably with the best in the United States.

In Havana, for the nine years ending with 1898, the average annual death-rate was nearly 47 in every 1,000 of population. In that year (1898) there were 21,252 deaths in the city. In twelve months this had been cut down to 8,153, which was further reduced to 6,102 in 1900, and 5,720 in 1901. Finally, the scourge of yellow fever has been practically wiped out. A record of the 45 years preceding 1901 shows an average of more than 751 deaths in every 1,000 cases, ranging from 2,058 in one season down to 51 in the best. American science and common sense sanitation have left for 1901 a record of but 18 deaths.

Surely the United States has redeemed its pledges and retired from the island with honor.

